# microRNA-150 Regulates Mobilization and Migration of Bone Marrow-Derived Mononuclear Cells by Targeting *Cxcr4*

**DOI:** 10.1371/journal.pone.0023114

**Published:** 2011-10-19

**Authors:** Nobuko Tano, Ha Won Kim, Muhammad Ashraf

**Affiliations:** Department of Pathology and Lab Medicine, University of Cincinnati, Cincinnati, Ohio, United States of America; Brigham and Women's Hospital, United States of America

## Abstract

The interaction between chemokine receptor type 4 (CXCR4) and its ligand, stromal cell-derived factor (SDF)-1, plays an important role in stem cell mobilization and migration in ischemic tissues. MicroRNAs (miRs) are key regulators of stem cell function and are involved in regulation of stem cell survival and differentiation to adopt different cell lineages. In this study, we show that ischemia inhibits the expression of miR-150 in BM-derived mononuclear cells (MNC) and activates its target *Cxcr4* gene. Our results show that miR-150/CXCR4 cascade enhances MNC mobilization and migration. By using mouse acute myocardial infarction (MI) model, we found that MNCs in peripheral blood (PB) were increased significantly at day 5 after AMI as compared to control group and the number of CXCR4 positive MNCs both in bone marrow (BM) and PB was also markedly increased after MI. Analysis by microarray-based miRNA profiling and real-time PCR revealed that the expression of miR-150 which targets *Cxcr4* gene as predicted was significantly downregulated in BM-MNCs after MI. Abrogation of miR-150 markedly increased CXCR4 protein expression suggesting its target gene. To show that miR-150 regulates MNC mobilization, knockdown of miR-150 in BM-MNCs by specific antisense inhibitor resulted in their higher migration ability *in vitro* as compared to scramble-transfected MNCs. Furthermore, *in vivo* BM transplantation of MNCs lacking miR-150 expression by lentiviral vector into the irradiated wild type mice resulted in the increased number of MNCs in PB after AMI as compared to control. In conclusion, this study demonstrates that ischemia mobilizes BM stem cells via miR-150/CXCR4 dependent mechanism and miR-150 may be a novel therapeutic target for stem cell migration to the ischemic tissue for neovascularization and repair.

## Introduction

Ischemic heart disease is a leading cause of death worldwide. As a result of an insufficient blood supply of the heart muscle by coronary occlusion, loss of viable cardiomyocytes and the reduction of cardiac output can be induced during AMI phase. BM-derived mononuclear cells including endothelial progenitor cells (EPCs) play an important role in the maintenance of vascular integrity [1], [2]. Since MNCs/EPCs are able to differentiate into mature endothelial cells and promote repair of damaged endothelium, they are attractive target for the repair of ischemic tissue [3]–[5]. MNC/EPC number and function are closely associated with coronary endothelial function, and reduced levels of circulating MNCs/EPCs have been shown to be independent predictors of atherosclerotic disease progression [6]. Therefore, sufficient MNC/EPC numbers, as well as the capacity to differentiate into mature endothelial cells are considered to be essential for myocardial functional recovery and infarct size reduction.

Interaction between stromal cell-derived factor-1α (SDF-1α, or CXCL12α) and its receptor CXC chemokine receptor 4 (CXCR4, or fusin/CD184) plays a key role in mobilization of vascular stem/progenitor cells [7]. As one of the strategies to rescue cardiac dysfunctions after AMI, the modification of CXCR4 expression in BM-derived stem cells has been investigated by using various BM-derived stem cells [8]. For instance, hypoxic preconditioning of cardiac stem/progenitor cells (cardiosphere-derived, Lin^−^ c-kit^+^ progenitor cells) upregulates CXCR4 expression and increases the recruitment of these cells into the ischemic myocardium, thereby reducing the infarct size and improving the cardiac function after MI [9]. In addition, intravenous delivery of mesenchymal stem cells (MSCs) overexpressing CXCR4 improves cardiac function and remodeling after MI, suggesting CXCR4 as an important therapeutic target for the treatment of cardiovascular diseases [10].

MicroRNAs (miRs) play an important role in the posttranscriptional regulation of target mRNA in a range of biological processes, including maintenance of stemness and modulation of mobilization, proliferation and differentiation. miRNAs are short (19–23 nucleotides), noncoding small regulatory RNAs that are loaded into the RNA-induced silencing complex, recognize the 3′-untranslated region (UTR) of target genes, and thereby regulate their expression by translational repression or mRNA degradation. [11], [12] Donahue and colleagues have previously profiled miR expression in response to Plerixafor (AMD3100, hematopoietic mobilizing agent) and granulocyte colony-stimulating factor (G-CSF), and found that these two agents mobilized different CD34 positive cell populations based on miR expression signatures, suggesting each miRs may regulate different group of BM cell mobilization [13]–[15]. However, the functional role of specific miRs and their targets for cell mobilization remains to be investigated.

Here we report CXCR4 expression as a target of miR-150 which is downregulated in BM-derived MNCs in response to AMI, leading to MNC mobilization and migration in PB.

## Materials and Methods

### Experimental Mouse model of AMI

C57BL/6 mice (female, 12 weeks old) were anesthetized by intrapertoneal injection of ketamine/xylazine (100 mg/kg and 20 mg/kg body weight, respectively). After endotracheal intubation and mechanical ventilation using Harvard Appratus MiniVent (Type 845), a left intercostal thoracotomy was performed to open the chest. Left anterior descending coronary artery was permanently ligated with 6-0 silk (Echicon) and the chest was closed. For post-mortem studies, we sacrificed animals using overdose of sodium pentobarbital. All experimental procedures were performed in accordance with the standard human care guidelines of the “Guide for the Care and Use of Laboratory Animals” and were approved by the Institutional Animal Care and Use Committee of University of Cincinnati (Protocol ID: 06-03-13-03), which conforms to National Institutes of Health guidelines.

### Isolation of MNCs

One ml of peripheral blood was harvested from control and AMI mice by aspiration from heart. Total BM cells were obtained by flushing the cavity of femurs, tibias, and ilium and bone fragments were filtered through a 40 mm filter. MNCs were isolated by density-gradient centrifugation using 1.083 g/ml Histopaque 1083 solution (Sigma-Aldrich). Briefly, Histopaque 1083 was added to centrifuge tube and whole blood was carefully loaded onto surface of the histopaque. Then, histopaque with whole blood was centrifuged at 400 g for exactly 30 minutes at room temperature. After discarding upper layer, the opaque inferface containing the MNC layer was transferred into a 15 ml tube and washed with PBS twice by centrifugation. After the final wash, MNCs were used for further study.

### miRs profiling with microarray

Total RNA samples obtained from PB- and BM-MNCs of control and AMI mice were sent to LC Sciences (Houston, TX) for miRs microarray profiling. Data were analyzed by LC Sciences with in-house developed computer programs. Intensity values were transformed into log2 scale, and fold changes were given in log2 scale. A *t*-test was performed between non-infarcted MNCs and infarcted MNCs from hearts, and statistical significance was considered at *p*<0.01.

### miRs isolation and detection

Total RNA, including miRs, was extracted by using *mir*Vana miR isolation kit (Ambion) in accordance with the manufacturer's instructions. miR-150 was detected by using RT^2^ miRNA First Strand Kit (SA biosciences) and specific miR-150 primers from QIAGEN were used for quantitative reverse transcription (qRT) PCR and real time PCR. Relative expression was calculated using the comparative Ct method (2^−[Δ][Δ]Ct^).

### Transfection of MNCs with miR inhibitor

To knockdown miR-150 in MNCs, transfection was performed with anti-miR-150 (miRCURY LNA microRNA inhibitor, Exiqon) by electroporation system according to the instruction of manufacture (Electroporator II, Invitrogen). Briefly, 10×10^6^ BM-MNCs were resuspended in 400 µl of DMEM and transferred into electroporation cuvette. For miR inhibitor electroporation, 100 nM of anti-miR-150 was added and the loaded cuvettes were placed on ice. Voltage on the Electroporator II was set at 660 V and an infinite internal resistance value was used. A blank cuvette containing PBS were discharged at least twice before electroporating cells. MNCs were electroporated two times and then transferred to culture dish. The electroporated cells were incubated at 37°C in 5% CO_2_ and examined 48 hr after electroporation using an appropriate assay.

### Migration assay

A total of 1×10^6^ BM-MNCs suspension in 250 µl serum-free EBM was placed in the upper chamber of Transwell Boyden chamber (Coster, 8 µm pore size, Sigma-Aldrich). Then the chamber was placed in 24 well culture dish containing 500 µl endothelial basal medium supplemented with 10% fetal bovine serum, singlequats and 100 ng/ml SDF-1 (Peprotech inc). After 24 hr of incubation at 37°C, the lower side of the filter was washed with PBS and fixed with 4% paraformaldehyde. Cells migrating to the lower chamber were stained with 0.1% crystal violet solution. Migration activity was evaluated as the mean number of migrated cells in three random microscopic fields (400×) per chamber.

### FACS analysis

PB- and BM-derived MNCs were resuspended in PBS containing 1% bovine serum albumin and were incubated for 30 min in the dark at 4°C with phycoerythrin (PE) -conjugated monoclonal CXCR4 antibody (R&D). Cells were washed three times by centrifuging with 1 ml of PBS for 5 min at 400 *g* before analysis.

### Western blot analysis

MNCs were lysed in lysis buffer, pH 7.4 [(in mM) 50 HEPES, 5 EDTA, 50 NaCl], 1% Triton X-100, protease inhibitors [(10 µg/ml aprotinin, 1 mM phenylmethylsulfonyl fluoride, 10 µg/ml leupeptin) and phosphatase inhibitors [(in mM) 50 sodium fluoride, 1 sodium orthovanadate, 10 sodium pyrophosphate]. The protein samples (40 µg) were electrophoresed using SDS-polyacrylamide gel and electro-immunoblotted as described. The specific antibody used for the detection of CXCR4 was purchased from ProSci Inc.

### miRs target gene prediction

Computational analyses to predict potential binding between 3′ UTR of target genes and miRNA was carried out with the following databases: microRNA.org (version 2010), TargetScanMouse (version 5.1) and MirBase (version 4). Only common targets were considered for experimental analysis.

### Lentivirus-mediated miR-150 inhibition in MNCs

miR-150 overexpressing vector and scramble plasmid were obtained from GeneCopoeia. Lentivirus overexpressing miR-150 was generated by using Lenti-Pac HIV Expression Packaging Kit (GeneCopoeia). Briefly, 2.5 µl of lentiviral miR-150 inhibitor expression plasmid/control plasmid, 5.0 µl of EndoFectin Lenti and EndoFectin Lenti reagent were added in Opti-MEM I, and formed the DNA-EndoFectine complex. Twenty min after incubating the complex at room temperature, the DNA-EndoFectine complex was added in the dish which 293Ta cells were plated in DMEM with 10% FBS and incubated in 5% CO_2_ at 37°C overnight. The culture medium was replaced with fresh DMEM with 5% FBS and 1/500 volume of the TiterBoost reagent to the culture medium and continued to be incubated. The virus pseudovirus-containing culture medium was collected 48 hr post transfection and concentrated after filtration. For the transduction of BM-MNCs with lentivirus, 10×10^6^ of MNCs was plated and 20 µl of virus suspension was added. The cells were placed for 2 hr at 4°C and then transferred to the plate in a 5% CO_2_ at 37°C for 48 h.

### BM transplantation model

Recipient C57BL/6 mice were exposed to a single sublethal dose of 5.5 Gy total-body γ-irradiation. BM-MNCs from donor mice were infected with Lentivirus overexpressing miR-150 as described above. Lentivirus transfected-MNCs were counted, and 5×10^6^ cells were injected into recipient mice via retro-orbital venous sinus at 6–8 hr after irradiation. Injection into the retro-orbital sinus was easier to perform but more invasive than using the tail vein [16], [17]. The mice were subjected to AMI and harvested MNCs from BM and PB at 5 days after AMI were analyzed as described above.

### Statistical analysis

All values were expressed as means ± SE. Comparison between two mean values was made by an unpaired Student two-tailed *t*-test. Statistical significance was accepted at *p*<0.05.

## Results

### AMI increases mobilization of BM-derived MNCs and CXCR4 positive cells into PB

**Figure 1:**
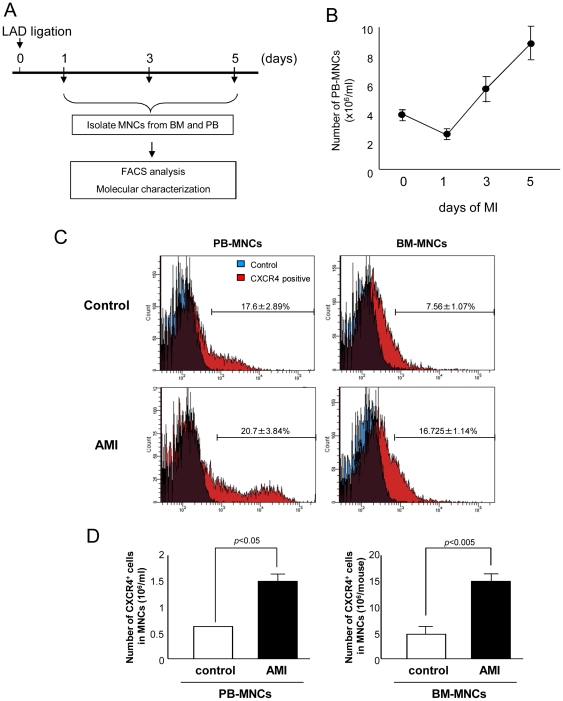
AMI increases the numbers of BM-MNCs and CXCR4 positive MNCs. (A) MNCs were isolated at 1, 3 and 5 days after left anterior descending artery ligation (LAD) and analyzed for molecular characterization. (B) The number of PB-MNCs decreased at one day after LAD ligation and increased gradually up to 5 days (n≥10 in each groups). (C) The percentage of CXCR4 positive cells in MNCs increased in both PB and BM after LAD ligation as compared to control. (n = 8 in control group and n = 6 in AMI group). (D) Densitometric analysis of CXCR4 positive MNCs counted after LAD ligation.

To examine whether AMI induces MNC mobilization from BM into PB, we assessed the number of MNCs in PB from mice at 1, 3 and 5 days after AMI and control mice as shown in Fig. 1A. The numbers of MNCs in PB were significantly decreased at day 1 after AMI (38.6% decrease) and markedly increased at day 3 (47.0% increase) and day 5 (110.7% increase) after AMI (Figure 1B). Furthermore, the percentage of CXCR4 positive MNCs isolated from both PB and BM of AMI mice was significantly increased (2.2 and 2.6 fold, respectively) as compared to those of non-infarcted mice (Figure 1C and D), indicating AMI induces mobilization of MNCs as well as CXCR4^+^ cells into blood circulation.

### miRs expression profiling in BM-derived MNCs from AMI mice

**Figure 2:**
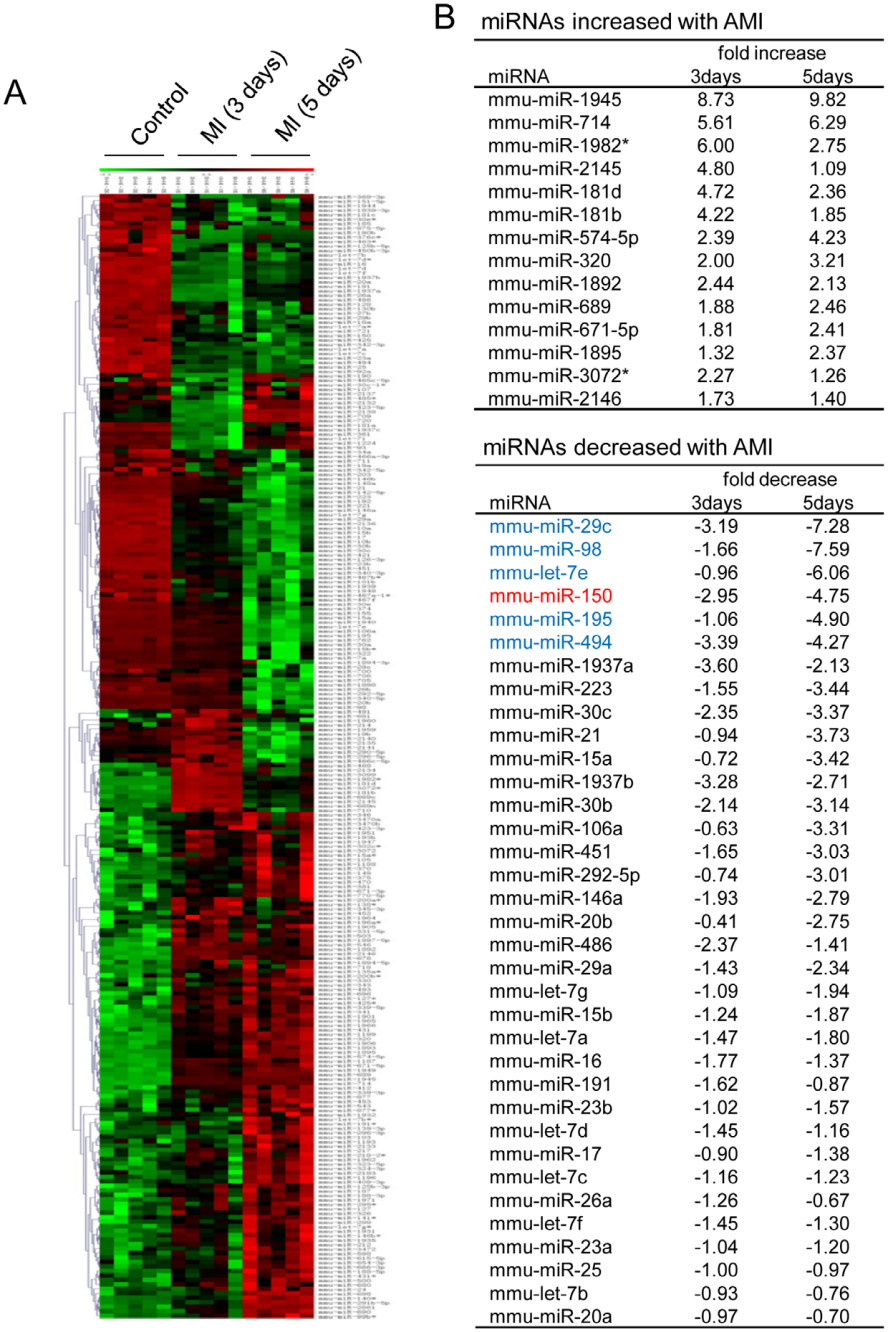
Microarray-based miRs expression profiling in BM-MNCs. (A) Gene tree represented on left and heat map on right of 15 experiments, respectively (n = 5). The color of the top bar shows the degree of expression (red; upregulated genes, green; downregulated genes). (B) The list of miRs whose expression was altered in BM-MNCs in response to LAD ligation.

To examine the potential participation of miRs in mobilization of MNCs, we performed miR profiling in BM-derived MNCs by using miRs microarray. As shown in Figure 2A and B, specific sets of miRs expression decreases more than 2 folds in BM-MNCs from AMI mice at 3 days as compared to control mice. These include miR-1937a (−3.6 fold), miR-494 (−3.39 fold), miR-29c (−3.19 fold), miR-150 (−2.95 fold), miR-486 (−2.37 fold), miR-30c (−2.35 fold) and miR-30b (−2.14 fold). Interestingly, miR-29c, miR-150, and miR-494 expression was further decreased more than 4 fold at 5 days (−7.28, −4.75, and −4.27, respectively), which led us to further study the role of these miRNAs in MNC mobilization.

### miR-150 regulates MNC migration by targeting *Cxcr4*

**Figure 3:**
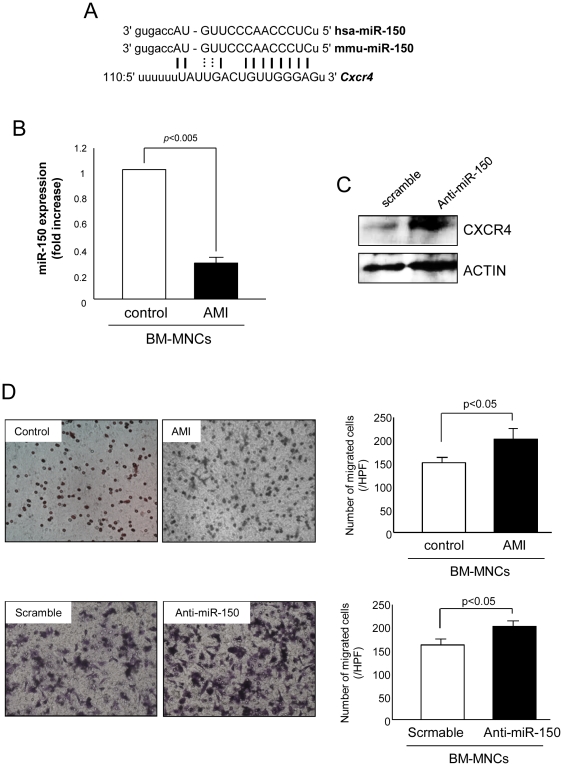
miR-150 targets Cxcr4 and regulates MNC migration. (A) A putative target site of miR-150 highly conserved in the *Cxcr4* mRNA 3′-UTR as predicted by computational analysis. (B) miR-150 expression was decreased in BM-MNCs after AMI as validated by real-time PCR. (C) CXCR4 protein expression in MNCs significantly increased by transfection with miR-150 inhibitor. (D) BM-MNCs isolated from mice with LAD ligation enhanced migration capacity of BM-MNCs in response to SDF-1α as evaluated by transwell migration system. Transfection of wild type MNCs with anti-miR-150 also increased the number of migrating cells.

Since miR microarray analysis revealed that miR-29c, miR-98/let-7 family, miR-150, miR-195 and miR-494 expression was significantly downregulated in BM-derived MNCs, we extensively examined databases for predicted targets of these miRNAs involved in MNC mobilization. Computational target prediction analysis indicated that *Cxcr4* is one of the consensus putative targets of miR-150 relevant to stem cell mobilization (Figure 3A). To verify microarray profiling results, we performed real time PCR and confirmed that miR-150 expression was markedly reduced in BM-derived MNCs from AMI mice (Figure 3B). As shown in Figure 3C, knockdown of miR-150 by anti-miR-150 markedly reduced CXCR4 protein expression in MNCs, indicating CXCR4 is a putative target of miR-150 in MNCs. To investigate the role of miR-150 in migration of MNCs, we performed *in vitro* cell migration assay by using Boyden Chamber transwell system. MNCs isolated from BM of AMI mice exhibited the higher number of migrated cells in response to SDF-1α as compared to control MNCs (Figure 3D, upper). Interestingly, knockdown of miR-150 by transfection with anti-miR-150 significantly increased the number of migrated MNCs when compared to scramble transfection, suggesting miR-150 is critically involved in MNC migration (Figure 3D, bottom).

### Knockdown of miR-150 enhances mobilization of MNCs *in vivo*

**Figure 4:**
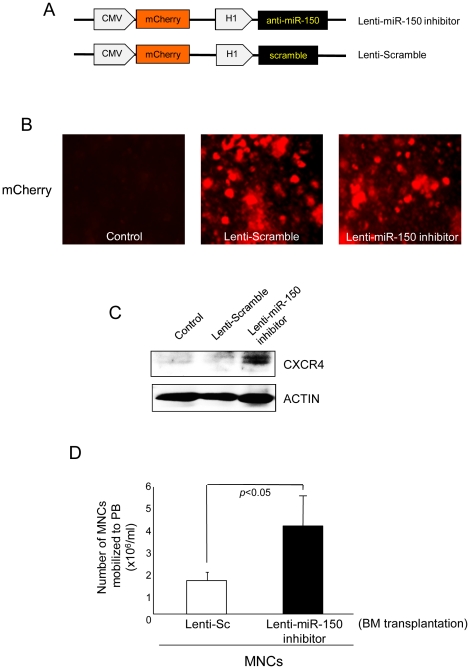
Lentivirus-mediated knockdown of miR-150 augments CXCR4 expression and mobilization of MNCs. (A) Structures of Lenti-miR-150 inhibitor and Lenti-Sc which contains mCherry reporter gene (red signal), (B) Titeration and transduction efficiency of lentivirus-mediated miR-150 inhibition in MNCs was evaluated by mCherry signal under microsope. (C) Lentivirus-mediated knockdown of miR-150 elevated CXCR4 protein expression in MNCs. (D) Number of BM-MNCs mobilized to PB was markedly increased in peripheral circulation of BN transplantation of mice which received MNCs lacking miR-150.

To further examine the involvement of miR-150 in stem cell mobilization *in vivo*, we performed mouse BM transplantation experiments by using irradiated mice and lentiviral miR-150 inhibitor. Lentivirus containing mCherry (red, reporter) was generated by co-transfection of 293Ta cells with Lentiviral vectors and miR-150 inhibitor or scramble (Figure 4A). We produced Lentivirus containing anti-miR-150 and miR-scramble and transduced to MNCs isolated from healthy mice. Lentivirus transduction efficiency was confirmed by red signal of mCherry under microscope. Figure 4B showed approximately >90% of MNCs expressed mCherry at 2 days of Lentivirus infection. In addition, knockdown of miR-150 in MNCs by lentiviral vector significantly increased CXCR4 expression (Figure 4C). Interestingly, we found that *in vivo* transplantation of MNCs lacking miR-150 expression (^anti-miR-150^MNCs) into the irradiated wild type mice resulted in increased number of MNCs in PB released from BM as compared to that of MNCs transducing scramble (^Sc^MNCs), indicating that miR-150 plays a critical role in MNC mobilization in BM through *Cxcr4* regulation (Figure 4D).

## Discussion

It has been reported that transplantation of BM-MNCs in patients with ischemic heart failure reduces major adverse cardiovascular events after MI and improved cardiac function [18], [19]. Several experimental studies suggest that most likely, multiple distinct cell populations of the BM-MNC are capable of contributing to neovascularization and differentiation into cardiac myocytes. MNCs systematically mobilized from BM can home to the sites of ischemic tissues such as infarcted myocardium, ischemic hindlimb, and the mechanism of MNCs homing to ischemic tissues has been extensively investigated. miRs are critically involved in many biological processes in health and disease including cardiovascular diseases. Although biological relevance of miRs in many stem cell functions is reported, the involvement of miRs in stem cell mobilization and migration is unknown.

In this study, we reported here for the first time that the expression of miR-150 was downregulated in BM MNCs in response to myocardial ischemia with simultaneous induction of CXCR4 protein expression. These molecular events led to enhanced mobilization and migration of BM MNCs. In our microarray-based miR profiling data indicated that miR-29c, miR-150, and miR-494 expression in BM-MNCs was significantly decreased by 4 fold at 5 days after AMI and the major objective of this study was to determine *Cxcr4* as a putative target gene of miR-150. The interaction of SDF-1α/CXCR4 plays a crucial role in stem cell mobilization. SDF-1α expression is upregulated by ischemia resulting in the recruitment and homing of hematopoietic stem/progenitor cells expressing CXCR4 receptor to ischemic sites [20]. Indeed, intramuscular injection of the chemoattractant chemokine SDF-1α has recently been shown to increase the number of incorporated EPCs and to improve neovascularization *in vivo* [21]. CXCR4 is a G protein-coupled 7-transmembrane receptor and plays crucial role in embryonic development as well as in maintaining the stem cell niche. Our data showed the numbers of PB-MNCs as well as CXCR4 positive MNCs were increased by AMI and identified miR-150 as a key regulator of BM-MNC mobilization by targeting *Cxcr4.*. Knocking down miR-150 by transfection with its inhibitor in MNCs exhibited the higher response to SDF-1α in migration capacity as compared with scramble-transfected MNCs. Furthermore, lentivirus-mediated knockdown of miR-150 significantly increased CXCR4 expression in MNCs and transplantation of these cells into BM of wild type mice dramatically increased MNC mobilization from BM to PB, indicating that miR-150 is critically associated with stem cell mobilization by regulating *Cxcr4*.

miR-150 is abundantly expressed in monocytes [22] which is altered under various conditions including the immune response and tumorigenesis [23], [24]. Therefore, miR-150 is being studied for its participation in addressing different aspects of cellular functions with special focus on its role in mobilization of monocytes. In this regard, *c-Myb*, *a* transcription factor related to endothelial cell migration and B cell differentiation, is being tipped as a potential target gene of miR-150 [22], [25]. Microvesicle-based exogenous miR-150 delivery to human microvascular endothelial cells resulted in downregulation of c-MYB expression and enhanced their migration ability *in vitro* [25]. Although these data support our findings to imply miR-150 in cell mobilization, the discrepancy between the two studies may be attributed to; 1) different cell types (endothelial cell *vs* stem cell), 2) cell maturity (mature vs progenitor cell), 3) exogenous vs endogenous expression of miR-150, 4) disease type (atherosclerosis *vs* myocardial ischemia), all of which might have led to the different results between these studies. Another research group has identified a molecular cascade (*PLZF* transcription factor/miR-146a/CXCR4) controlling normal megakaryopoiesis which involved enhanced expression of *PLZF* and inhibition of miR-146a transcription [26]. They demonstrated downregulation of miR-146a elevated the translation of the target *CXCR4* mRNA. In this regulatory loop, miR-146a acted as an effector of *PLZF* and mediated its control activity on *Cxcr4* translation. Taken together, these studies indicated the functional significance of miR-150 in immune system and disease control and, warrant further investigations to elucidate the functional role of miR-150 in angiomyogenesis mediated by mobilized MNCs.

In conclusion, our results demonstrated that miR-150 downregulation by AMI enhanced CXCR4 expression, leading to enhanced BM-MNCs mobilization and migration. Thus, miR-150 may be a novel therapeutic target for stimulating BM cell mobilization to ischemic tissue for participation in the repair process.
